# Use of consumer wireless devices by South Africans with severe communication disability

**DOI:** 10.4102/ajod.v5i1.202

**Published:** 2016-02-19

**Authors:** Juan Bornman, Diane Nelson Bryen, Enid Moolman, John Morris

**Affiliations:** 1 Centre for Augmentative and Alternative Communication, University of Pretoria, South Africa; 2 Centre for Augmentative and Alternative Communication, Temple University, United States; 3 Shepherd Center, Atlanta, United States

## Abstract

**Background:**

Advancements in wireless technology (e.g. cell phones and tablets) have opened new communication opportunities and environments for individuals with severe communication disabilities. The advancement of these technologies poses challenges to ensuring that these individuals enjoy equal access to this increasingly essential technology. However, a paucity of research exists.

**Objectives:**

To describe the nature and frequency with which South African adults with severe communication disabilities have access to and use wireless devices, as well as the types of activities for which wireless devices are used.

**Method:**

Survey research was conducted with 30 individuals who use augmentative and alternative communication (AAC) technology using the Survey of User Needs Questionnaire developed in the United States, and localized to the South African context.

**Results:**

All participants, despite their limited education, unemployment and low economic status, owned and/or used mainstream wireless devices. Slightly more than half of the participants (53.3%) needed adaptations to their wireless devices. Advantages of using wireless devices were highlighted, including connecting with others (through using text messaging, social networking, making plans with others, sharing photos and videos with friends), for leisure activities (e.g. listening to music, watching videos, playing games), and for safety purposes (e.g. to navigate when lost, using the device when in trouble and needing immediate assistance).

**Conclusion:**

These wireless devices offer substantial benefits and opportunities to individuals with disabilities who rely on AAC in terms of independence, social participation, education and safety/security. However, they still do not enjoy equal opportunity to access and use wireless devices relative to the non-disabled population.

## Introduction

At the turn of the century Africa had only 15 million cell phone subscribers. That number grew to 387.7 million over the next decade. By 2011, Africa became the second largest mobile phone market after Asia (Dlamini Zuma 2014).

According to the Pew Research Center (2015), today cell phones (portable telephones that use wireless cellular technology) are as commonly used in South Africa as they are in the United States. Smartphones (those that run complete operating systems and that can access the internet and applications with features such as calendars, media players, GPS navigation, and web browsing) are not as widely used (Bryen & Moolman 2015). Slightly more than 34% of South Africans own these devices compared to 64% in the United States (Pew Research Center 2015).

The rapid growth of wireless technologies in South Africa has bypassed the earlier need for landlines. Today, landline penetration in sub-Saharan Africa is close to zero with only 6% of South Africans reporting having a working landline phone in their household (Pew Research Center 2015). Consequently, wireless devices, including cell phones and smartphones, are a critical means of communication and information access in South Africa.

Access to these wireless technologies has dramatically increased communication, expanded commerce, and improved access to information via the Internet. These technologies have a tremendous potential for all South Africans, including those with disabilities. According to the Center for an Accessible Society (2014), mobile cellular technologies offer remarkable possibilities to enhance the quality of life and increase the independence of people with disabilities. For example, persons with severe communication disability can now log in and order groceries online, shop for appliances, research health questions, participate in online discussions, catch up with friends or make new ones.

However, there is a paucity of information that describes the use of wireless technologies by those individuals with severe communication disabilities who cannot rely on their natural voice to meet their daily communication needs. These individuals typically have little or no functional speech (Bornman 2015). Although no prevalence figures are available for South Africa, prevalence studies shows that approximately 1.3% of the world population cannot rely on their natural speech to meet their daily communication needs (Beukelman & Mirenda 2013). This translates to 702 000 South Africans if the current South African population size of 54 million people is used (Statistics South Africa 2014). Communication disability results in these individuals being restricted in their participation in all aspects of life – education, employment, family life and citizenship.

Augmentative and Alternative Communication (AAC) technology involves multiple modes of communication, including specialized devices with synthesized or digitized speech. Wireless devices, such as cell phones, smart phones and tablets can also act as communication devices (Alzrayer, Banda & Koul 2014). They allow for voice input, text messages, picture capturing, e-mailing, internet access and gaming (York & Fabrikant 2011). The availability of social media, such as Facebook, LinkedIn and Twitter on many of these wireless devices has further expanded communication opportunities for these individuals (Beukelman & Mirenda 2013; Caron & Light 2015).

Research from the United States reported that individuals with severe communication disabilities and who have access to wireless technologies use them for much the same activities as the larger disability community (Caron & Light 2015; McNaughton & Light 2013). Unfortunately, these individuals have less access to these technologies, lagging behind the larger disability community who are already lagging behind their peers without disability (Morris & Bryen 2015).

Recognizing the importance of these wireless technologies, the United Nations identified them as a critical success factor for the inclusion of persons with disabilities in their post-2015 agenda (G3ict 2013). The UN further estimated that such technologies have the potential for making significant improvements in the lives of the 15% of the world population who have some form of disability. Similarly, 14 articles of the United Nations Convention on the Rights of Persons with Disabilities mandate or address access to information and communication technologies (G3ict 2013). In South Africa, as worldwide, these mainstream wireless devices can improve the lives of South Africans with disabilities, especially those who have severe communication disabilities. However, little is known about their access to and use of these technologies due to a paucity of research in this area. Therefore the question remains as to the degree to which South African adults with severe communication disabilities have access to and use wireless devices, as well as the type of activities for which wireless devices are used.

## Research method and design

### Research aims

The primary aim of this research was to describe the nature and frequency with which adults who have severe communication disabilities and who use AAC systems in South Africa have access to wireless devices, and to describe the activities for which these devices are used. Four sub-aims informed the primary research aim:

1. Describe the types and use of specialized assistive devices used by participants;
2. Determine wireless device ownership and describe the source(s) used to select these devices;
3. Describe the use, importance, and satisfaction with wireless devices, the types of activities participants engage in, as well as their frequency; and
4. Describe participants’ recent experiences with their primary wireless devices.

### Design

A descriptive survey design (McMillan & Schumacher 2010) was used to describe the responses of 30 literate South African adults with severe communication disabilities who use AAC systems. Information was obtained through the completion of the Survey of User Needs (SUN 4) questionnaire (Morris, Mueller & Jones 2014) that was adapted specifically for the South African context.

### Materials

SUN was launched in the United States in 2002 and has been updated three times in order to keep up with the rapid pace of technological change (Morris *et al.* 2014). The most recent version (SUN 4) was launched in 2012 and can be viewed at http://www.wirelessrerc.org/content/projects/sun-overview.

SUN 4 covers five main areas of inquiry. Part 1 focuses on relevant biographical data. The focus of Part 2 is participants’ abilities and difficulties, as well as types of assistive devices that they use. Part 3 focuses on participant’s use of mainstream wireless devices, and Part 4 on activities for which the wireless devices are used, and how often they are used. Part 5, which focuses on wireless service providers, was omitted in this research, as most of the participants did not know the answers to these questions.

## Validity and reliability

In order to enhance the content validity of SUN 4, interviews were conducted during its development with subject matter experts in the wireless device industry and regulatory agencies, accessibility and assistive device experts, advocates for people with disabilities and people with disabilities themselves. A few items were adapted from other established survey research projects, including the National Health Interview Survey conducted by the U.S. Centers for Disease Control and Prevention and the Pew Research Center’s on-going research on wireless device use (Duggan & Smith 2013).

For the purposes of this study, SUN 4 had to be slightly modified for the South African context. Five specific adaptations were made: (1) ethnicity was adapted to include the accepted South African ethnic groups; (2) highest level of education had to include education at a special school; (3) household income was measured in South African Rand, and a distinction was made between households with an income below R60 000.00 per annum and those above, as individuals who earn below R60 000.00 are exempt from paying personal income tax (Tax Statistics 2008) and hence form the lower socio-economic status group; (4) examples of mobile technologies specific to the South African context were included; and (5) cost of apps was changed to South African Rand.

Reliability of the data was enhanced by using trained research assistants who assisted participants to complete the questions (Babbie & Mouton 2001). Following this, all participants were asked if there was anything additional that they would like to add.

### Participant recruitment

Three recruitment strategies were used. The first was e-mail recruitment from participants and alumni of the Fofa Project, which is a unique programme to empower adults with severe communication disabilities who use AAC, and which has been presented annually since 2005 at the University of Pretoria. *Fofa* is a Sotho word, meaning *to fly* or *to* *soar*, and Fofa participants actively engage in a week-long seminar designed to teach skills such as how to effectively communicate using their AAC devices and then applying these newly acquired skills to everyday situations (http://www.up.ac.za/centre-for-augmentative-alternative-communication/article/56192/about-fofa). This first recruitment strategy yielded 15 participants.

Second, 11 potential participants who use AAC systems and who were known to the researchers were contacted via e-mail. This resulted in seven participants agreeing to participate, surpassing the accepted 50% response rate (Babbie & Mouton 2001). Third, three institutions for adults with disabilities were contacted and eight potential participants identified. All provided consent and completed the survey.

Unfortunately, the three means of participant recruitment is likely to have resulted in a biased sample. Sample bias is likely due to an over-representation of participants who have stronger literacy, higher education and greater supports.

### Data analysis

Descriptive statistics formed the basis for the data analysis (McMillan & Schumacher 2010). Information from SUN 4 was coded in Survey Monkey and frequencies and percentages were calculated for purpose of analysis.

### Participant description

A total of 30 literate South African adults with disability who have little or no functional speech and who use AAC systems participated in this research. Demographic information is provided in Table 1. Only 13.3% of the participants completed the survey independently. The majority of participants were white men between the ages of 18 and 49 years. Most participants had attended special schools, lived in urban areas with someone else, were unemployed and had a low socio-economic status.

**TABLE 1:** Participant demographic information (*N* = 30).

Demographic Information	%
**Method of completing the survey**	
Research assistant assisted	40.3
Parent or other family member assisted	33.3
Independently	13.3
Primary caregiver (paid or unpaid) assisted	6.7
Friend assisted	3.3
**Gender**	
Male	63.7
Female	36.7
**Age**	
18–29 years of age	46.7
30–49 years of age	40.0
50 years of age and older	13.3
**Race/Ethnicity**	
White people	66.7
Black people	33.3
**Gross annual household income**	
Less than R60 000.00 per year	60.0
More than R60 000 per year	40.0
**Education**	
Completed Grade 9	13.3
Completed high school (Grade 12)	13.3
3–4 years post school	6.7
5 or more years post school	10.0
Attended special school	36.7
Completed special school Grade 10	6.7
Completed special school Grade 12	13.3
**Employment status**	
Employed full or part time	16.7
Retired	3.3
Unemployed	80.0
**Living arrangements**	
Urban area	70.0
Suburban area	20.0
Rural area	10.0
Lives alone	6.7

Table 2 shows that all participants reported that they had difficulties speaking so that other people could understand them. Additionally, the majority had motor disabilities (i.e., difficulties walking, climbing stairs and using their arms, hands, and fingers). Some reported that they had difficulty concentrating, remembering and making decisions (26.7%) and some experienced frequent worry, nervousness and anxiety (16.7%). Few had sensory difficulties such as difficulty seeing (13.3%) and difficulty hearing (6.7%).

**TABLE 2:** Type of difficulty experienced by participants (N = 30).

Type of difficulty	%*
Difficulty speaking so people can understand	100.00
Other (running; balancing; sitting and/or standing; dressing; chewing and/or eating; toileting)	76.7
Difficulty walking and climbing stairs	73.3
Difficulty using hands and fingers	70.0
Difficulty using arms	60.0
Difficulty concentrating, remembering, making decisions	26.7
Frequent worry, nervousness or anxiety	16.7
Difficulty seeing	13.3
Difficulty hearing	6.7

* Percentages add to more than 100%, because several participants experienced multiple difficulties.

### Procedure

Participants received information about the research and were requested to complete an informed consent letter. The participants recruited though Fofa completed their surveys at the University of Pretoria, whilst participants recruited via e-mail completed their surveys at home, and participants recruited from institutions completed their surveys at their respective institutions. Most were individually assisted by trained research assistants in completing the survey, with a small percentage completing it independently. The same instructions were given throughout, using the questions as stipulated in the SUN 4 survey. On average it took the participants between 60 and 90 minutes to complete the survey.

#### Ethical considerations

Ethics approval was obtained from the Faculty of Humanities Ethics Committee, University of Pretoria (Reference number GW20150315HS). Three main ethical considerations were respected. Firstly, the principle of voluntary participation was paramount, since participants were not pressured into participation. It was reiterated that they could withdraw from the research at any time without any negative consequences. Secondly, informed consent was obtained (Campbell *et al.* 2010). All participants received written information about the nature of the research, informing them that it posed neither risks nor benefits. Information and consent letters were written at a fourth-grade reading level using the Flesch-Kincaid grade level computer analysis (McClure 1987) to ensure readability. They were also informed about how data would be analysed and distributed and were offered the option to receive a copy of the article following publication. Finally, confidentiality (Smith 1995) is seen in that data were coded to protect participants’ individual data and all identifying information was removed from this article. No identifying information was made available to anyone who was not directly involved in the research.

## Results

Results are described according to the four sub-aims of the research.

### Use of specialized assistive devices

Given the participants’ multiple disabilities detailed in Table 2, it is not surprising that a variety of specialized assistive devices were used. All used some form of AAC, consisting of speech-generating AAC devices (56.7%), text-to-speech software (46.7%), AAC communication boards (40.0%), or a combination of these. Many used mobility devices, especially wheelchairs (70%). Very few used specialized devices to aid their sensory disabilities, such as hearing aids or screen readers.

### Ownership of wireless devices and source(s) used to select these devices

All participants reported that they owned a primary wireless device, such as a cell phone, smartphone or tablet. A primary wireless device was defined as the device they used most, whereas the secondary device was the one they used secondarily, either for particular tasks or in certain contexts.

Table 3 shows that 23.3% owned a basic phone, whilst two-thirds owned a smartphone, including Android-powered smartphones (23.3%), Blackberry devices (20%) and Windows-powered smartphones (20%). iPhones were used less frequently. Additionally, 10% of respondents reported owning a tablet: iPad (7%), or Android tablet (3%).

**TABLE 3:** Ownership of primary and secondary wireless device(s) (N = 30).

Type of primary wireless device owned	% of ownership	Type of secondary wireless device owned	% of ownership
Owns a primary wireless device	100	Owns a secondary wireless device	33.3
Basic cell phone	23.3	Basic cell phone	0.0
Android smartphone	23.3	Android smartphone	10.0
Apple iPhone	3.3	Apple iPhone	0.0
Blackberry smartphone	20	Blackberry smartphone	0.0
Windows-powered smartphone	20	Windows-powered smartphone	0.0
Android-powered tablet	3.3	Android-powered tablet	6.7
Apple iPad	6.7	Apple iPad	10.0
Other	0.0	Other	6.7

Approximately one-third (33.3%) of the participants owned a secondary wireless device such as an Android Smartphone, iPhone, and an Android-powered tablet or iPad. However, due to space limitations, this article focuses only on primary wireless devices.

Participants were asked about use of landlines in their place of residence. Less than half (46.7%) reported that they had access to a working landline telephone at home and only one person reported that he used it to make or receive calls.

Participants reported using a variety of sources to select their wireless devices. One-third (33.3%) bought their wireless devices based on recommendations from a friend, family member or healthcare professional. An additional 30% obtained them as a donation or had them on loan. Less frequently, participants based their decisions on television, radio or magazine advertisements (13.3%); on a salesperson’s advice (13.3%); on the features for persons with disability shown on the product label (13.3%); or on website information from either wireless service companies (6.7%) or device manufacturers (6.7%).

### Use, importance, satisfaction, ease and changes made to primary wireless devices and type and frequency of engagement

Table 4 shows that most participants indicated that they used their wireless device for personal use only (66.7%), with a smaller percentage using these technologies for both professional and personal use (26.7%). Almost all rated the importance of their device as either ‘very’ or ‘somewhat important’, irrespective of the type of device. Similarly, 83% were either ‘very satisfied’ or ‘somewhat satisfied’ with their device.

**TABLE 4:** Use, importance, satisfaction, ease, as well as changes made to current primary wireless device (N = 30).

Aspect of current primary wireless considered	%
**Purpose of use**	
Personal use	66.7
Both professional and personal use	26.7
Emergencies only	3.3
Professional use (work or school)	3.3
**Importance of use**	
Very important	83.3
Somewhat important	13.3
Not very important	3.3
**Satisfaction**	
Very satisfied	50.0
Somewhat satisfied	33.3
Neither satisfied not dissatisfied	10.0
Very dissatisfied	6.7
**Ease of use**	
Very easy to use	43.3
Easy to use	30.0
Somewhat hard to use	16.7
Cannot use it without help	10.0
**Changes/additions made**	
No changes or additions	46.7
Physical accessories added, such as protective skin or case; headset; Bluetooth device; screen overlay; lanyard; stylus	36.7
Software added, such as third party text-to-speech; screen reader; screen magnifier; app store downloads	26.7
Assistive devices added, such as head switch; EMG switch; AAC device; neck loop; TTY	6.7
Improvised solutions, such as hand strap, Velcro, wheelchair mount	6.7
Other, such as larger font, different screen glass for head pointer, protective screen	6.7

Table 4 also shows that 73.3% of the participants reported that their wireless device was either ’very easy’ or ’easy to use’. However, 26.7% reported that it was ’somewhat hard to use’ or that they ’couldn’t use it without help’. More than half of the participants had made some additions or adaptations to their ‘off-the-shelf’ devices. These included adding commercially available physical accessories (36.7%) or adding software (26.7%). Improvised solutions or adding of assistive devices and other solutions were used to a lesser degree (6.7%). These data suggest that off-the-shelf, specialized, and improvised adaptations were needed to use their wireless devices.

Participants were asked about their wireless activities. Results are described in terms of how frequently they use their primary wireless device (Table 5).

**TABLE 5:** Frequency of primary wireless device usage irrespective the type of activity (N = 30).

General usage of device	%
Several times a day	66.7
About once a day	10.0
3 to 5 days a week	6.7
1 or 2 days a week	6.7
Less often than weekly	3.3
Don’t know	3.3

This section was further expanded by asking about the types of activities that participants engaged in with their primary wireless device (Table 6), frequency of engagement in these activities as well as their use of social networking sites and apps (Table 7).

**TABLE 6:** Participants’ wireless activities and their frequency of use (N = 30).

Activities primary wireless device is used for	%
Text messaging	80.0
Keeping a directory of contacts	73.3
Sharing photos or video online	66.7
Listening to music	60.0
Social networking (e.g. Facebook, LinkedIn, Twitter)	56.7
Web browsing	56.7
Voice calling	50.0
Keeping a calendar of appointments	43.3
Downloading applications	43.3
Watching videos	40.0
Email	36.7
Playing games	33.3
Navigating and way finding (GPS)	23.3
Using voicemail	13.3
Recording voice notes or reminders	10.0
Shopping	3.0
Video calling	3.3
Other	13.0

**TABLE 7:** Frequency of participants’ wireless activities: Text messaging, voice calling, social media and downloading apps (N = 30).

Activity	%
**Text messaging: Text messages made and received on average day**	
None	10.0
Less than 5	30.0
Between 5 and 9	6.7
Between 10 and 19	10.0
Between 20 and 29	6.7
Between 30 and 49	3.3
Between 50 and 79	16.7
More than 80	6.7
Other (many/don’t know)	10.0
**Frequency of using social networking sites**	
Never	33.3
Several times a day	43.3
About once a day	6.7
3–5 days a week	3.3
1 or 2 days a week	3.3
Every few weeks	3.3
Less that monthly	3.3
**Social networking sites with profiles**	
Facebook	80.0
LinkedIn	13.3
Twitter	10
Other (e.g. We chat)	3.3
**Phone calls made and received on an average day**	
None	43.3
Less than 5 phone calls	50.0
Between 5 and 10 phone calls	0.0
Between 10 and 15 phone calls	3.3
More than 15 phone calls	3.3
**Number of apps used on a typical day**	
Cannot download apps	23.3
None	10.0
1 or 2 apps	43.3
3 to 5 apps	13.3
6 to 10 apps	6.7

Overall, the majority reported using their primary wireless device either several times each day (66.7%) or at least once daily (10%). Text messaging was used by most participants (80.0%) with a variable degree of frequency. Text messaging was followed by keeping a directory of contacts (73.3%), sharing photos or videos online (66.7%), and listening to music (60.0%). More than half used their wireless devices for social networking, with 67% noting that they have a profile on Facebook, Twitter and/or LinkedIn. Frequency of use of these social working sites ranged from several times a day (43.3%) to not at all (33.3%), with Facebook being the favoured social networking site (80%). Only 12% did not use social networking sites on a daily basis. Regarding a social network profile, many respondents had at least one social networking profile (46.7%).

Web-browsing was also used by 56.7%, and half of the participants used their wireless device for voice calling despite their difficulties with spoken language. Using the calendar for appointments was reported by 43% and watching videos by 40.0%. Sending and receiving emails (36.7%), playing games (33.3%), and navigating to find their way (23.3%) was also used with varying degrees of frequency. Only 13.3% of the participants used the voicemail option, and 10.0% used voice notes or reminders. ‘Other’ activities included typing stories and poems (6.7%), listening to the radio (3.3%) or as an augmentative communication device (3.3%).

Table 7 also shows that 43.3% of the participants stated that they used one or two apps on their wireless devices. Some of the apps that the participants used were Facebook, WhatsApp, BBM, Proloquo2Go, Music, Photos, Super sport, Microsoft office, Viber, TeamViewer, Quick support and YouTube. Less than half of the respondents (43.3%) stated that they had never paid for an app and only downloaded apps that were free. Another 23.3% stated that their wireless device could not download apps, whilst 23.3% stated that, although their device could download apps, they did not download any. A further 6.7% each said that they spent R10.00 or less, between R10.00 and R50.00 or more than R200.00 on an app, or that they did not know what the cost of their apps were.

Finally, participants were asked whether there was anything that they would like an app to do that the current apps could not. Some responded that they would like an app that constantly linked their wireless device to their computer so that they did not have to re-launch the TeamViewer app and get it all linked up again each time they switched it on. Another reported wanting a Calculator, Instagram, Text-to-speech app, WhatsApp with text-to-speech, Link everything e.g., phone, laptop, TV (replace remotes) and watch DVDs.

### Recent experiences with their primary wireless devices

Participants were asked about their experiences with their wireless devices during the past 30 days. Table 8 shows that participants used their devices most frequently to make plans with others (66.7%), for entertainment (63.3%), and to get information that they needed right away (46.7%).

**TABLE 8:** Recent experiences with primary wireless device (N = 30).

Recent experiences with primary wireless device	%
I used my wireless device to make plans with others	66.7
I used my wireless device for entertainment or when I was bored	63.3
I used my wireless device to get information that I needed right away	46.7
I was in a situation where I had trouble doing something because I didn’t have my wireless device with me	36.7
I had difficulty entering a lot of text	30.0
I was frustrated because my wireless device took too long to use	30.0
I was in an emergency situation where having my wireless device really helped	26.7
I used my wireless device to get directions while outside of my home or office	23.3
I had difficulty reading because the screen or the text was too small, or the screen reader couldn’t read it out loud	16.7
I turned my wireless device off for a period of time to get a break from using it	13.3
I pretended to use my wireless device to avoid interacting with people around me	10.0

Some participants identified having frustrations using their device, including that their devices took too long to download something (30%), having difficulty entering a lot of text (30%), and having difficulty reading something on their device because the screen was either too small or the screen reader could not read the text aloud (16.7%). In contrast, some respondents reported a certain reliance on their devices: 30% reported having trouble doing something because they did not have their device with them; 26.7% reported being in an emergency situation when their device helped them. Finally, 13% indicated that they turned their device off for a period of time, just to get a break from using it.

## Discussion

The findings of this research yielded important information about the use of mainstream wireless devices by a small, non-representative sample of South African adults with severe communication disabilities. Foremost, it must be noted that all participants in this research own and use mainstream wireless devices, such as cell phones, smartphones, or tablets. This is a surprising finding given that many of the participants had limited education, were unemployed, and came from households with low socio-economic status. However, it should be remembered that 30% had received their devices as gifts or donations. The fact that 100% of participants owned a wireless device might be attributed to the fact that they all had severe communication disabilities, and that these mainstream devices are often used as AAC devices in developing countries due to their affordability (McNaughton & Light 2013) and availability (Shane *et al.* 2012). This is in contrast to recent research in the US, where it was found that individuals who use AAC (similar to the participants in the current research) own cell phones and other wireless devices at substantially lower rates than their peers with other disabilities (Wireless RERC 2014). In fact, earlier US research reported that this population had limited access to cell phones (Bryen, Carey & Friedman 2007).

Most of the participants had multiple physical difficulties along with significant speech disabilities that may be expected to place significant barriers to their use of mainstream wireless technologies. The results showed that just over half of the participants (53.3%) made some adaptations to their devices to allow them to successfully access and use their devices. The findings also showed that the participants chose to use cell phones rather than landlines despite the fact that cell phones were difficult to use for individuals with significant physical and communication disabilities. The fact that the participants employed in this research were ’AAC and device wise’ as they had been exposed to AAC, could have contributed to their wireless technology usage.

It has been hypothesized that persons with severe communication disabilities often have limited social networks to begin, which may act as a barrier to cell phone use (Bryen & Moolman 2015). However, in this research two-thirds of the participants (66.7%) reported that they used their wireless devices to make plans with others, whilst more than half of the participants (56.7%) used their devices for social networking (e.g. Facebook, LinkedIn, Twitter). This finding might thus suggest that owning a wireless device could in fact be seen as an enabling factor for social networking, which in turn might reduce the participants’ vulnerability for abuse due to social isolation (Brown 2004). It has been documented that limited social interaction and subsequent isolation and dependence on others gradually carves away self-esteem on the basis of the disability, resulting in emotional deprivation, a dependent relationship with the personal assistant, feelings of helplessness and powerlessness as well as ignorance about violence (Bornman 2015).

Apart from this protective function, a recent US study that focussed on social media showed similar results to the findings of the current study when the authors described the benefits for persons who use AAC as connecting them with other individuals, making them feel typical, making communication easier, gaining independence and getting help (Caron & Light 2015). They also found potential employment benefits. Unfortunately that was not seen in this South African research.

Earlier AAC studies had revealed that almost two-thirds of young adults with severe communication disabilities felt that, although their AAC devices were useful, they were also ‘uncool’ or boring, did not fit their self-image, and did not produce the desired benefit in terms of interaction control (Clarke *et al.* 2001). In contrast, wireless devices have many potential benefits for individuals with severe communication disabilities, including increased awareness and social acceptance (Lorah *et al.* 2013) as well as greater functionality and interconnectivity (McNaughton & Light 2013). This was also evident in the current research when considering the wide range of activities for which wireless devices were used, as well as the frequency with which they were used.

## Limitations of the study

A methodological limitation was the relatively small sample size. In addition, participants were not randomly selected, since most were current participants or alumni of Fofa, and familiarity with the researchers might have resulted in participants being eager to help and therefore acting in a socially desirable manner by responding positively to items in the survey. Familiarity with AAC devices might also have rendered them ’device wise’ and hence a different sample might have yielded different results.

Furthermore, the participants in this research did not reflect the racial/ethnic distribution of South Africa. There was an over-representation of white South Africans and an under-representation of other ethnic groups in the sample. The under-representation of black participants most likely reflect the inaccessibility and disproportionate access for these South Africans to schools (and hence literacy instruction), as well as other social services.

Additionally, literacy was a requirement for participation in the research, and this might have skewed the results, as low literacy levels exist in South Africa amongst people with disabilities (Integrated National Disability Strategy 1997). No prevalence figures exists for South Africa, but the most commonly quoted figure is UNESCO’s figure estimating a global literacy rate of 3% worldwide across the disability spectrum (Groce & Bakshi 2009). If non-literate individuals had participated, their use of wireless technology might have been substantially different. These demographic factors suggest that the findings of this research cannot be generalized to the broader South African population of people who use AAC.

## Conclusions and recommendations

This research provides evidence suggesting that wireless technology has the potential to benefit the lives of South Africans with severe communication disabilities in a variety of life activities, particularly in as far as social networking, safety and leisure activities, as well as direct communication is concerned. This is a particularly important finding given that wireless technologies are ubiquitous in the general population of South Africa (Pew Research Center 2015). Furthermore, these mainstream mobile technologies can also serve the functions of specialized AAC technologies – this is another important and positive implication of the findings of the study, since these mainstream technologies are comparatively more powerful, compact, and have a longer battery life, which is essential for communicating without worrying to stop and recharge a device, as is often the case with specialized AAC devices (Alper & Haller 2015. Additionally, as Caron and Light (2015) and Light and McNaughton (2014) suggest, social media via networked mobile technologies give individuals with significant communication disabilities opportunities to ‘increase, maintain, or improve’ their own communication in everyday contexts, be they synchronous or asynchronous conversations, face-to-face or from a distance, and among others with or without disabilities. Recent research has already demonstrated the benefits of the use of social media by individuals with severe communication disabilities in the United States (Caron & Light 2015).

Future research would benefit from a larger and more representative sample to ensure that the findings of this study are not limited to this sample of individuals who have significant communication disabilities and who use AAC. Additionally, future research should focus on how the interconnectivity between extant specialized AAC devices and mainstream wireless devices can be enhanced and, more importantly, how universal design principles can be applied when developing mainstream wireless devices in order to reduce the number of changes/adaptations that were made to allow access to and use of the wireless devices, especially as they relate to the full spectrum of fundamental information and communication activities in the 21st century. In conclusion, our findings provide empirical support for the position of Foley and Ferri (2012) that technology should be conceived of as a global, accessible and inclusive concept, not one that requires a qualifier based on whether or not the potential user has a disability.
